# Comparison of surface- and voxel-based registration on the mandibular ramus for long-term three-dimensional assessment of condylar remodelling following orthognathic surgery

**DOI:** 10.1259/dmfr.20210499

**Published:** 2022-02-25

**Authors:** Michael Boelstoft Holte, Henrik Sæderup, Else Marie Pinholt

**Affiliations:** 1 3D Lab Denmark, Department of Oral and Maxillofacial Surgery, University Hospital of Southern Denmark, Esbjerg, Denmark; 2 Department of Regional Health Research, Faculty of Health Sciences, University of Southern Denmark, Esbjerg, Denmark

**Keywords:** Three-Dimensional Imaging, Cone-Beam Computed Tomography, Computer-Assisted Surgery, Temporomandibular Joint, Orthognathic Surgery

## Abstract

**Objectives:**

The purpose of the present study was to validate and compare the accuracy and reliability of surface- and voxel-based registration on the mandibular rami for long-term three-dimensional (3D) evaluation of condylar remodelling following Orthognathic Surgery.

**Methods:**

The mandible was 3D reconstructed from a pair of superimposed pre- and postoperative (two years) cone-beam computerized tomography scans and divided into the condyle, and 21 ramal regions. The accuracy of surface- and voxel-based registration was measured by the absolute mean surface distance of each region after alignment of the pre- and postoperative rami. To evaluate the reliability, mean absolute differences and intraclass correlation coefficients (ICC) were calculated at a 95% confidence interval on volumetric and surface distance measurements of two observers. Paired t-tests were applied to statistically evaluate whether the accuracy and reliability of surface- and voxel-based registration were significantly different (*p* < 0.05).

**Results:**

A total of twenty subjects (sixteen female; four male; mean age 27.6 years) with class II malocclusion and maxillomandibular retrognathia, who underwent bimaxillary surgery, were included. Surface-based registration was more accurate and reliable than voxel-based registration on the mandibular ramus two years post-surgery (*p* < 0.05). The interobserver reliability of using surface-based registration was excellent, ICC range [0.82–1.00]. For voxel-based registration, the interobserver reliability ranged from poor to excellent [0.00–0.98]. The measurement error introduced by applying surface-based registration for assessment of condylar remodelling was considered clinical irrelevant (1.83% and 0.18 mm), while the measurement error introduced by voxel-based registration was considered clinical relevant (5.44% and 0.52 mm).

**Conclusions:**

Surface-based registration was proven more accurate and reliable compared to voxel-based registration on the mandibular ramus for long-term 3D assessment of condylar remodelling following Orthognathic Surgery. However, importantly, the performance difference may be caused by an inappropriate reference structure, proposed in the literature, and applied in this study.

## Introduction

Three-dimensional (3D) cone beam computed tomography (CBCT) has become the imaging modality of choice over two-dimensional (2D) cephalometric evaluation for assessment of condylar remodelling.^1–8^ An inherent shortcoming of the manual landmark-based cephalometric analysis is the accumulation of landmark identification errors, which are shown to range from 0.02 to 2.47 mm.^9–11^ These errors influence the measurements and make cephalometric analysis costly in terms of manual processing time. Hence, image superimposition and registration techniques have gained popularity in automated 3D assessment of surgical outcome and anatomical growth.^12^

Three different techniques exist for superimposition and registration of 3D images: landmark-, surface- and voxel-based registration. Landmark-based registration still requires manual identification of a set of minimum three landmarks in each corresponding image^13^ and has been shown to be inferior to surface- and voxel based registration.^14–18^ Surface-based registration aligns two 3D surfaces using the iterative closest point algorithm.^19,20^ Voxel-based registration uses the grayscale of the voxels to align two 3D volumetric images to the best superimposition.^21,22^

Surface- and voxel-based registration have previously been validated for different purposes^19,20,23–25^ and were compared for 3D assessment of surgical outcome in adult orthodontic subjects,^26^ following orthognathic surgery^27^ and for evaluation of growing subjects.^18^ The two registration methods were found to be reliable and accurate.^18,26,27^ Differences between the two registration methods were statistically insignificant and were unlikely to have any clinical importance.^18,26,27^ However, the performance of the registration methods has not been compared for evaluation of condylar remodelling. These methods are dependent on the reference structure, the region or volume of interest, which is an important observer-dependent input parameter. Most often the anterior cranial base is used as a stable reference structure, unaffected by growth and surgery, and has been validated and shown high accuracy.^28^

The anterior cranial base was used in previous voxel-based registration studies for evaluation of condylar remodelling.^29–34^ However, due to postoperative positional changes of the mandible, it is impossible to distinguish condylar remodelling from condylar displacement. Hence, condylar remodelling cannot be accurately assessed using the cranial base as a reference, and thus, reference structures on the mandible have been suggested.^23,25,34–41^

Recently, Verhelst et al performed a systematic review on 3D CBCT analysis protocols for condylar remodelling following orthognathic surgery, concluding that an anatomical region not prone to postoperative changes should be used for registration.^1^ Subsequently, Verhelst et al suggested and validated a protocol including the coronoid process, the ramal part between the mandibular notch and the posterior border of the ramus as stable structures following bilateral sagittal split osteotomy (BSSO).^25^ The protocol was validated using voxel-based registration of immediate (one week) and short-term (six months) postoperative CBCT. The authors advised further studies in order to confirm that the modified ramus reference structure does not remodel following surgery.^25^ Mutual image information of long-term postoperative CBCT scans used for the registration of the ramus is less similar due to bone remodelling processes. This subsequently affects the performance of the registration, and thus, the 3D assessment. Hence, the validity of surface-and voxel-based registration using the modified ramus as a reference structure for long-term (≥two years) evaluation of condylar remodelling is not yet proven.

Multiple recent systematic reviews conclude that condylar remodelling should be taken into account as a potential postsurgical complication following orthognathic surgery.^1,42,43^ However, available literature is characterised by low level of evidence and great heterogeneity with regard to its incidence and quantification. Hence, additional long-term research using validated 3D assessment techniques is needed for more definitive conclusions.^1,42,43^

The purpose of the present study was to validate and compare the accuracy and reliability of surface- and voxel-based registration on the mandibular rami for long-term 3D evaluation of condylar remodelling. The null hypothesis was: H_0_: the accuracy and reliability of surface- and voxel-based registration on the mandibular rami two years post-surgery are not significantly different in subjects with and without condylar resorption.

## Methods and materials

Permission was granted by the Institutional Ethics Committee – University Hospital of Southern Denmark, Esbjerg (21/33871). As the study comprised retrospective material, none of the subjects were exposed to any extra radiation and no extra examination has been performed to acquire additional information.

### Study sample

This study was based on pre- and postsurgical (two years) CBCT-scans from a study sample diagnosed with maxillary and/or mandibular growth disturbances, who underwent a combined bilateral sagittal split osteotomy (BSSO) and Le Fort I procedure at the Department of Oral and Maxillofacial Surgery, University Hospital of Southern Denmark, Esbjerg, Denmark. The study sample was randomly selected, such that half of the patients were diagnosed with postoperative condylar resorption and the other half was without.

Inclusion criteria: age range of 18–65 years; diagnosis indicating a combined BSSO and Le Fort I osteotomy; availability of patients’ pre- and postoperative (two years) CBCT scans. Exclusion criteria: previous history of oral and maxillofacial surgery, the presence of craniofacial anomaly or syndrome.

### Image acquisition

The CBCT images were acquired using an i-CAT scanner, version 17–19 (Imaging Sciences International, Hatfield, PA): 120 kVp; 5 mA; 7 s exposure time; Field-of-View (FOV) = 23×17.8 cm (768 × 768×576 voxels); isotropic voxels of 0.30 mm. The patients were scanned in an upright natural head position. The condyles were seated in centric relation (CR) and the preoperative occlusion in CR was fixed by a wax-bite, however, without a wax-bite at the postoperative CBCT scanning acquisition. The CBCT data were exported in DICOM format and imported into Mimics^®^ 24 (Materialise NV, Leuven, Belgium).

### Surgical technique

The surgery was performed in general anesthesia as a mandible-first procedure. After BSSO using a Obwegeser-Dal Pont split of the ramus with a Hunsuck modification, the distal mandibular segment was positioned using an intermediate splint and was fixated bilaterally to each proximal segment with two 2.0 four hole osteosynthesis plates (KLS Martin, Tuttlingen, Germany). A position pin (Medicon^®^ eG, Tuttlingen, Germany) placed at the Glabella was used as an external reference point to establish the planned vertical position of the upper incisors. Following the Le Fort I osteotomy, the maxilla was segmented into three pieces, in segmental cases, and the tooth-bearing segments were positioned into the final occlusion without the use of a splint, and subsequently fixated using two 2.0 Y-plates anteriorly and two L-plates posteriorly (KLS Martin, Tuttlingen, Germany). All osteotomy sites were grafted.

### Image segmentation and 3D reconstruction of the mandible

**Figure 1.**
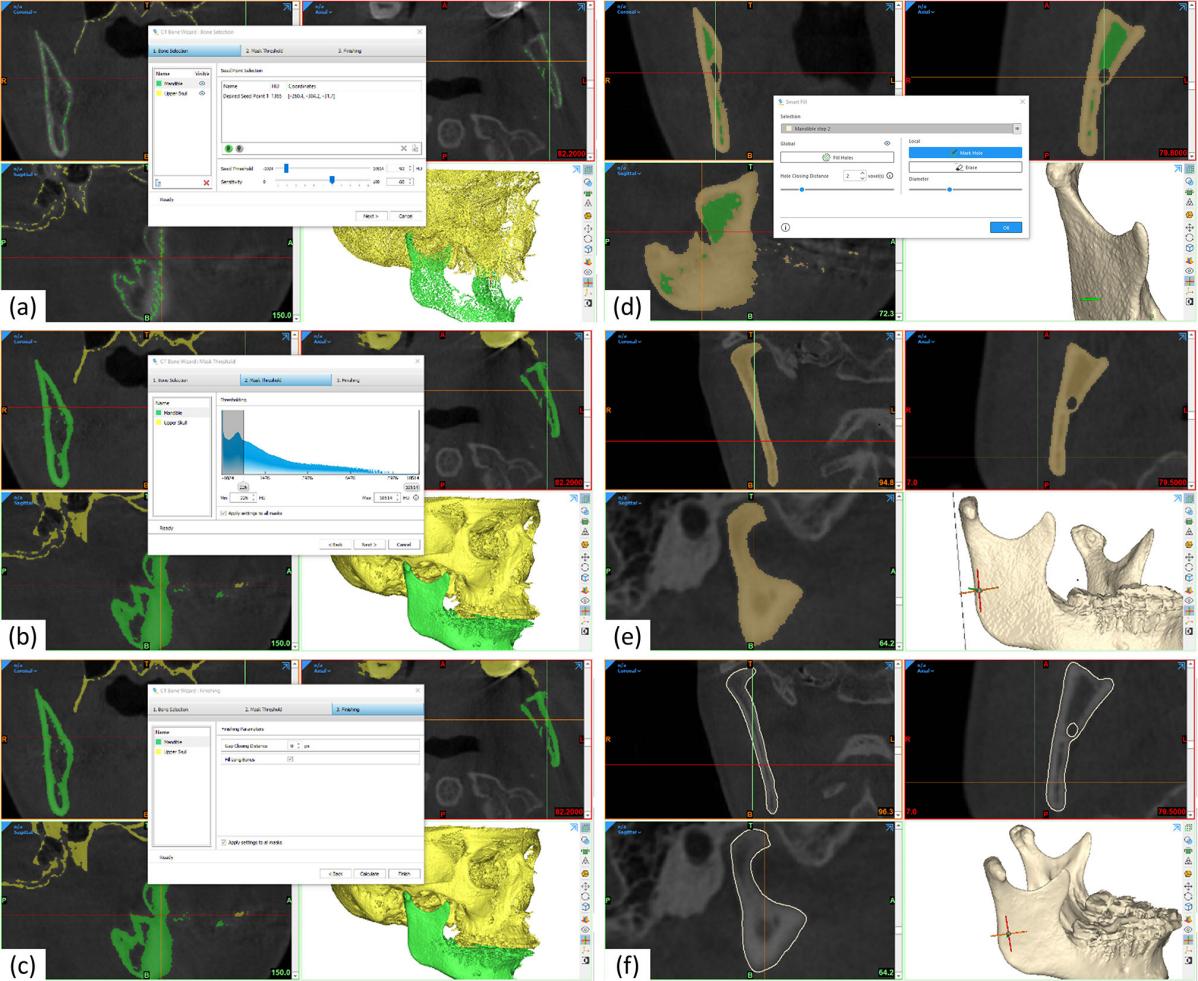
Illustration of the segmentation process. (**a**) CT bone wizard: selecting seed points and settings. (**b**) CT bone wizard: setting mask threshold. (**c**) CT bone wizard: finishing parameters. (**d**) Smart filling of bone segments. (**e**) Final mask. (**f**) Final 3D part.

The mandible was semi-automatically segmented using Mimics^®^ 24 (Materialise NV, Leuven, Belgium). For this purpose, the CT Bone Wizard was used (Figure 1). The CT Bone Wizard is a more advanced bone segmentation tool compared to a standard global threshold. First, a seed point, seed threshold and growing sensitivity were specified in Hounsfield Unit (HU) (Figure 1a). Next, the threshold of the mask was chosen (Figure 1b). The minimum threshold was set to 226 HU, the predefined lower threshold for CT bone. It should be noted that the accuracy implied by CT HU values is not available in CBCT. Finally, the finishing parameters were chosen (Figure 1c). The gap closing distance was set to 0 pixels, and instead the Smart Fill function was used to ensure that the rami bone segments were correctly filled (Figure 1d). Any remaining noise was removed manually using the Edit Mask tool. The final mask and 3D reconstructed part are shown in Figure 1e, f, respectively. The 3D parts were constructed with the following configurations: voxel resolution (x-, y- and z-multiplier: 1); smoothing (iterations: 10, smoothing factor: 0.2); triangle reduction (reducing mode: advanced edge, tolerance: 0.0375 mm, edge angle: 10˚, iterations: 3).

### Surface- and voxel-based registration

**Figure 2.**
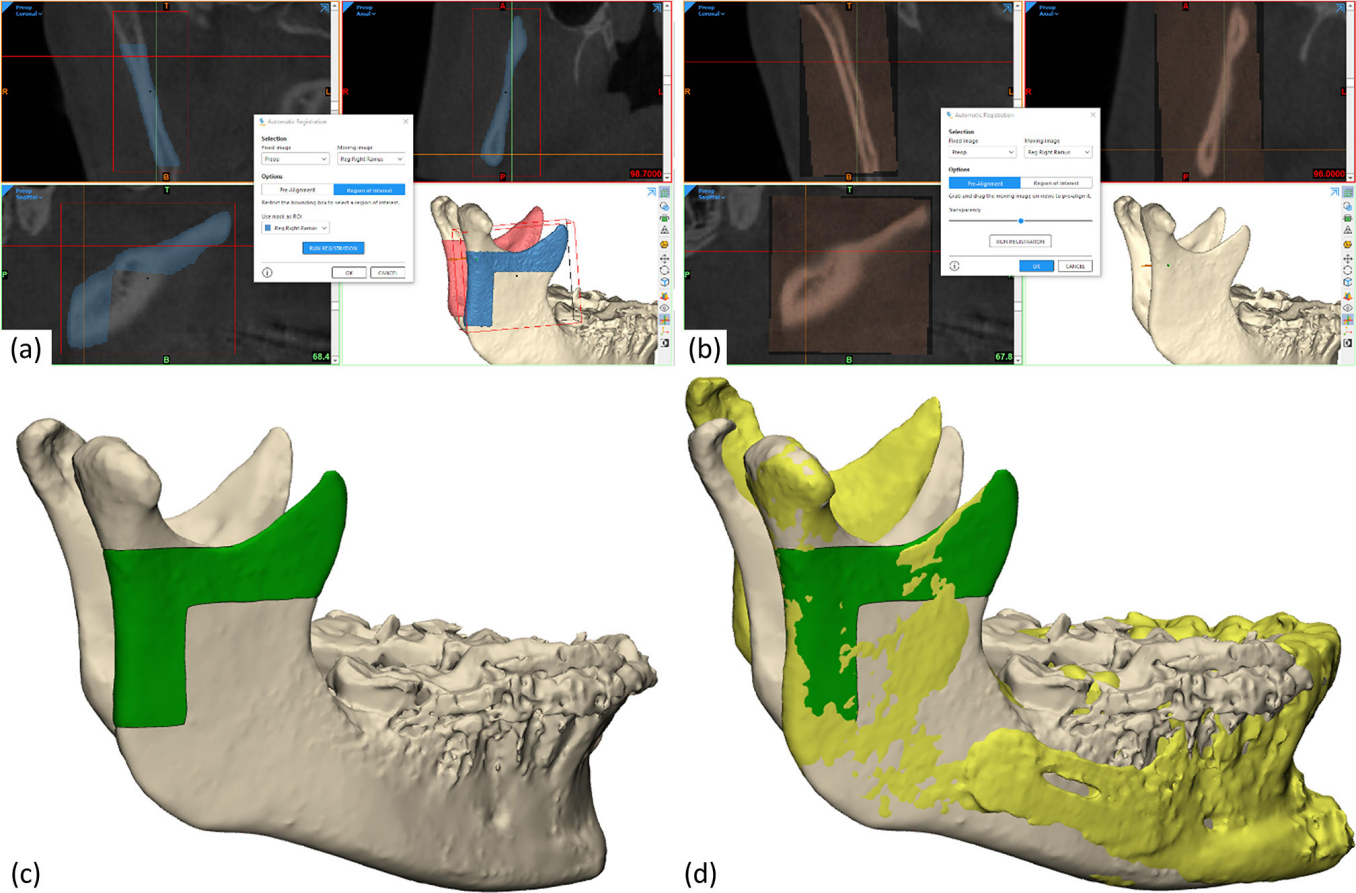
Surface and voxel-based registration on the right ramus. Voxel-based registration: (**a**) pre-alignment of CBCT scans and selecting the reference structure using a boundary box and a mask, (**b**) the resulting registration of the pre- and postoperative CBCT scans. Surface-based registration: (**c**) selecting the reference structure using a surface mask on the 3D part, (**d**) the resulting registration of the pre- and postoperative 3D objects. Note that the registration is performed on the right and left ramus, separately. Consequently, the the left postoperative ramus (in yellow) is not aligned.

For superimposing the pre- and postoperative CBCT scans, both voxel- and surface-based registration were applied to evaluate which method performed the best for superimposing the CBCT scans of the rami. The stable reference structures for registration recently proposed by Verhelst et al were chosen, containing the coronoid process, the ramal part between the mandibular notch and the posterior border of the ramus (Figure 2a).^25^ Voxel-based registration was performed in Mimics^®^ 24 (Materialise NV, Leuven, Belgium) using the Automatic Image Registration tool. Figure 2a, b shows the registration process from pre-alignment to selection of region of interest and the resulting registration result. The surface-based registration was performed using the Global Registration function (Figure 2c, d). The voxel- and surface-based registrations were performed for the right and left ramus, separately.

### Volumetric and surface distance measurements

**Figure 3.**
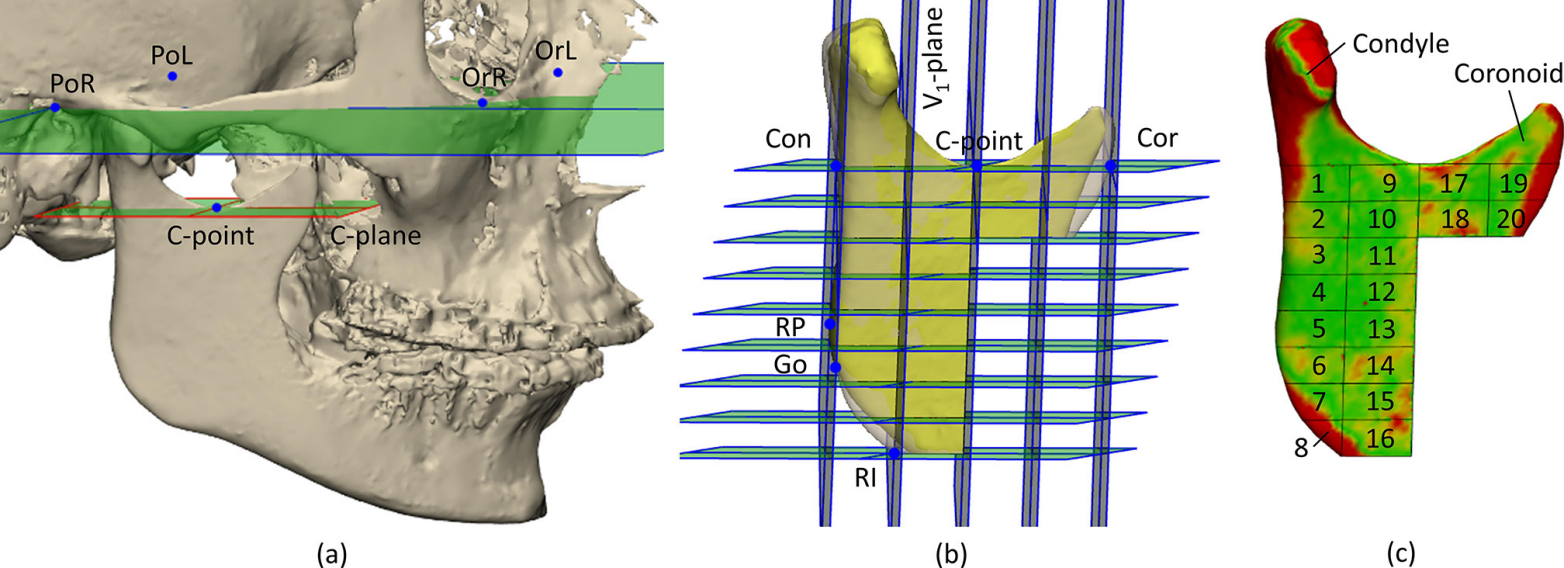
Analysis of morphological discrepancy between pre- and postoperative ramus regions. (**a**) Frankfurt- and C-plane. (**b**) Divisions of the ramus into the condyle, coronoid process and 20 subregions. (**c**) 3D color-coded distance map showing surface discrepancy in mm.

The analysis was implemented in 3-matic^®^ 16.0 (Materialise NV, Leuven, Belgium) and the workflow was automated using Python scripting. The only manual input required was the pre-operative anatomical landmarks listed in Table 1. The steps for analysing the right and left ramus, separately, were as follow: the C-plane, centred in the pre-operative mandibular notch (C-point) and parallel to the Frankfurt plane, as defined by Xi et al,^8,29,30^ was used to separate the pre- and postoperative condyle and coronoid process from the remaining ramal part (Figure 3). The ramal part was divided into 20 regions based on cutting-planes defined by five ramal landmarks (Figure 3b): (i) The ramus plane defined by the Condor point (Con), Coronoid point (Cor) and the Gonion (Go). (ii) The posterior ramus plane going through the most posterior point of the ramus (RP) and Con, and perpendicular to the ramus plane. (iii) The anterior ramus plane centred in Cor and parallel to the posterior ramus plane. (iv) The inferior ramus plane defined by the most inferior ramus point (RI) and parallel to the C-plane. (v) From the posterior- to the anterior ramus plane, three intermediate parallel planes with equal spacing were defined. (vi) From the C-plane to the inferior ramus plane six intermediate parallel planes with equal spacing were defined. The volume and surface discrepancy of the condyle, the coronoid process and the 20 regions were computed and exported (Figure 3c).

**Table 1.** Definitions of 3D cephalometric landmarks

Landmark	Definition	Bilateral
Orbitale (Or)	The most inferior anterior point on the orbit’s margin.	R/L
Porion (Po)	The most upper point on the bony external auditory meatus.	R/L
Mandibular notch (C-point)	The most caudal point of the mandibular notch.	R/L
Condor point (Con)	The most posterior point of the mandibular ramus intersecting the C-plane.	R/L
Coronoid point (Cor)	The most anterior point of the mandibular ramus intersecting the C-plane.	R/L
Gonion (Go)	The most caudal and most posterior point of the mandibular angle.	R/L
Ramus posterior (RP)	The most posterior point on the ramus intersecting the ramus plane.	R/L
Ramus inferior (RI)	The most inferior point on the ramus intersecting the V_1_-plane.	R/L

L, left; R, right.

### Statistical analysis

**S**tatistical analysis of the data was performed in STATA^®^ 16.1 (StataCorp, College Station, TX). A sample size calculation for one sample correlation studies (ICC>0.6, power = 0.8, α = 0.05, and two raters) was performed for each group. To evaluate the reliability, two observers independently performed the assessment (MBH, HS). Intraclass correlation coefficients at a 95% confidence interval on measurements of the two observers were calculated for single measurements using a one-way random effect model. The accuracy of the two registration methods was measured in accordance to previous comparative studies,^26,27^ *i.e*. by the absolute mean surface distance after alignment of the pre- and postoperative rami. The accuracy and reliability measurements of the condylar and the ramal regions were summarised using mean absolute differences (MAD) and standard deviations (SD). Paired t-tests were applied to statistically evaluate whether the accuracy and reliability of surface- and voxel-based registration were significantly different (*p* < 0.05).

## Results

The statistical sample size calculation resulted in a required sample size of *n* = 20 rami in each group to obtain a statistical power of 0.8. Hence, 20 randomly selected post-pubertal patients (40 rami) in a database who met the inclusion- and exclusion criteria, were recruited, such that half of the patients (20 rami) were diagnosed with postoperative condylar resorption and the other half was without (20 rami). Sixteen female; four male; mean age 27.6 ± 8.0 years; skeletal class II malocclusion with mandibular retrognathia (sixteen subjects); maxillomandibular retrognathia (four subjects); anterior open bite (fourteen subjects); lateral open bite (one subject); deep bite (one subject); mandibular asymmetry (four subjects); vertical maxillary hyperplasia (one subject); treated with maxillomandibular advancement (eighteen subjects); mandibular advancement (two subjects); maxillary expansion (nineteen subjects) and supplementary genioplasty (five subjects).

### Reliability

**Figure 4.**
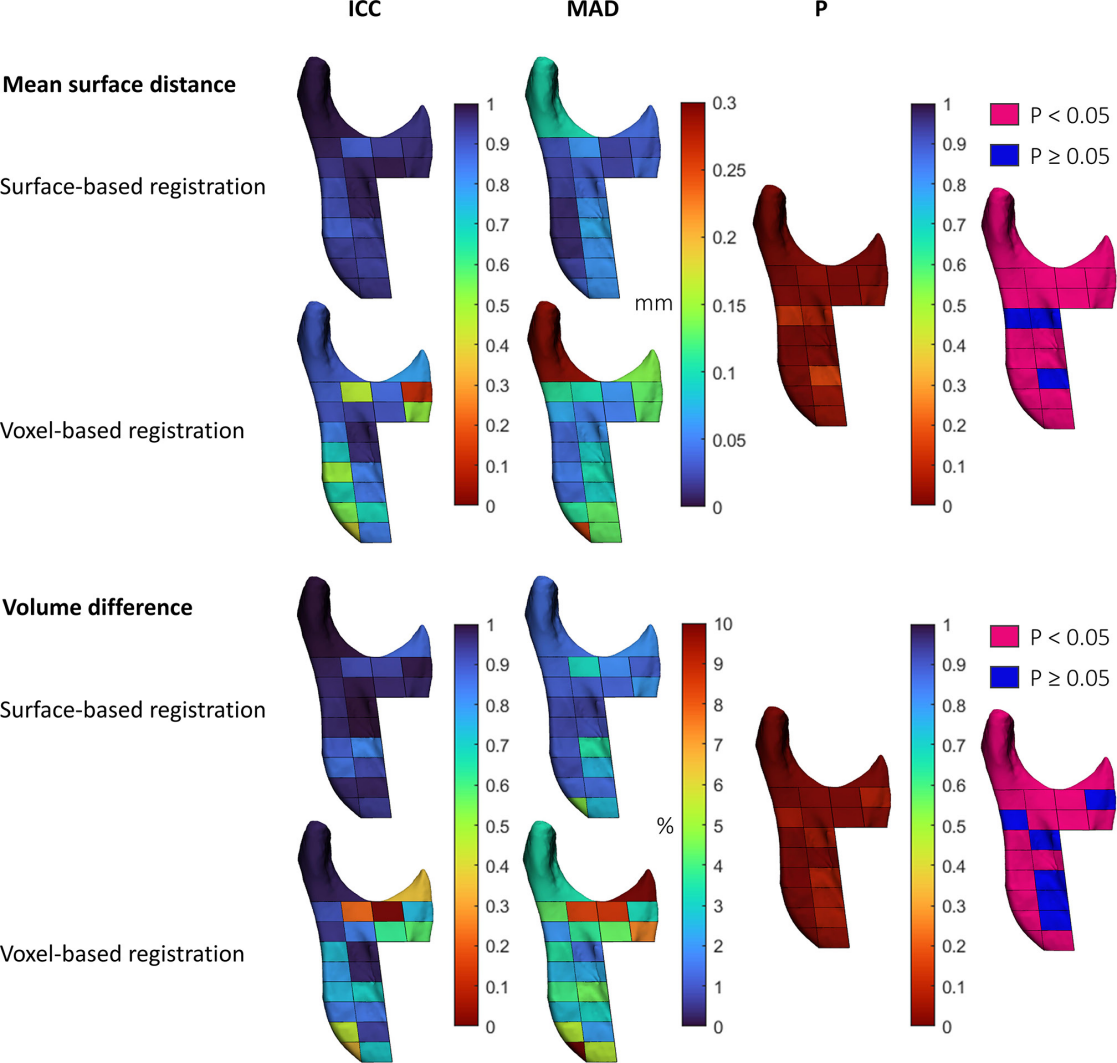
Illustration of the statistical results of comparing the reliability of the volumetric and surface distance measurements of the condylar and ramal regions produced by two observers using surface- and voxel-based registration in all subjects. Mangenta regions: surface-based registration statistically significant more reliable than voxel-based registration. Blue regions: no statistical significant difference between surface- and voxel-based registration. ICC: intraclass correlation coefficient, MAD: mean absolute difference, P: *p*-value.

**Figure 5.**
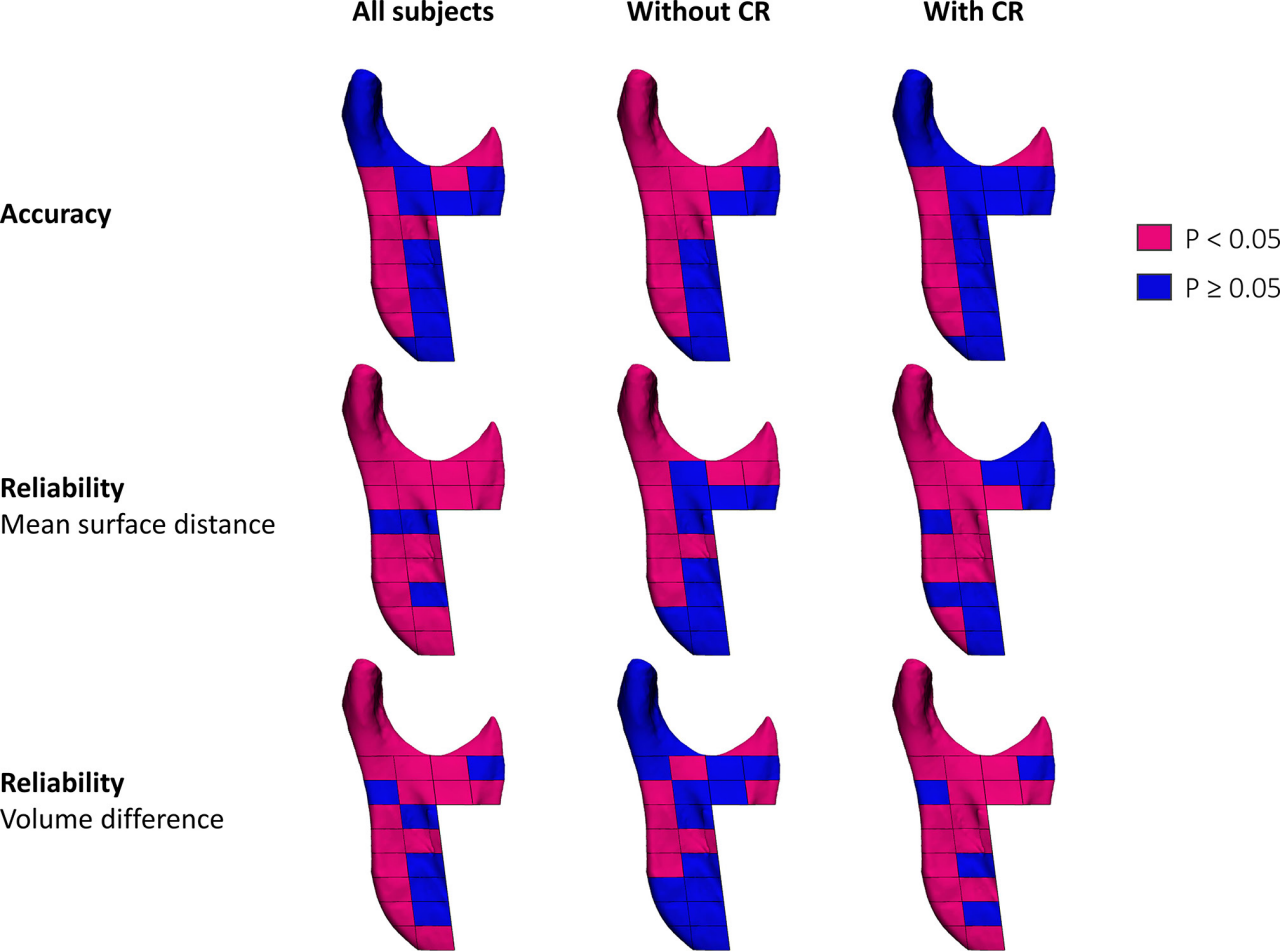
Illustration of the statistical significant difference of surface- and voxel-based registration for the condylar and ramal regions. Mangenta regions: surface-based registration statistically significant more accuracte/reliable than voxel-based registration. Blue regions: no statistical significant difference between surface- and voxel-based registration. CR, condylar resorption.

Surface-based registration was in general more reliable than voxel-based registration on the mandibular ramus. The superior reliability was significant for the repeated surface distance measurements of the condyle, the coronoid process and 17/20 ramal regions in all subjects. For the repeated volumetric measurements, the superior reliability was significant at the condyle, the coronoid process and 14/20 ramal regions in all subjects (Figure 4 and Table 2 in Supplementary Material 1). The reliability discrepancy between the two methods was more pronounced in subject with condylar resorption than in those without (Figure 5 and Table 3-4 in Supplementary Material 1).

The range of the MAD (SD) between the repeated surface distance measurements produced by two observers using surface- and voxel-based registration was [0.01 mm (0.00)–0.18 mm (0.16)] and [0.02 mm (0.01)–0.52 mm (0.43)], respectively. For the repeated volumetric measurements, the range of the MAD (SD) was [0.26% (0.31)–6.25% (4.08)] and [0.77% (0.90)–32.19% (22.85)], respectively (Table 2-4 in Supplementary Material 1). The MAD (SD) of the repeated condylar surface distance measurements using surface- and voxel-based registration in subjects with condylar resorption was 0.18 mm (0.16) and 0.52 mm (0.43), respectively. For volumetric measurements, the MAD (SD) was 1.83% (1.66) and 5.44% (3.82), respectively (Table 4 in Supplementary Material 1). Hence, the discrepancy between the condylar assessments using surface- and voxel-based registration in subjects with condylar resorption was 3.61% and 0.34 mm in favour of surface-based registration.

The interobserver reliability of the volumetric measurements and surface distances of the individual regions using surface-based registration in all subjects was excellent,^44^ ICC range [0.82–1.00] and [0.88–0.99], respectively. For voxel-based registration, the ICC range was poor to excellent [0.00–0.98] and [0.11–0.97], respectively. The low ICC values for voxel-based registration were observed at the coronoid process and the most distant ramal regions (Table 2 in Supplementary Material 1).

### Accuracy

Surface-based registration was in general more accurate than voxel-based registration on the mandibular ramus. The superior accuracy was significant for registration of the coronoid process and 9/20 ramal regions in all subjects (Figure 5 and Table 5 in Supplementary Material 1). Surface-based registration was also significantly more accurate for registration of the condyle, the coronoid process and 11/20 ramal regions in subjects without condylar resorption (Figure 5 and Table 6 in Supplementary Material 1).

The range of the MAD (SD) between the volumetric measurements and surface distances produced by the two registration techniques was [0.64% (0.61)–12.34% (10.19)] and [0.02 mm (0.02)–0.34 mm (0.37)], respectively. The largest differences between the two methods were observed in subjects with resorption at the condyle (2.52% (2.11) and 0.34 mm (0.37)), at the coronoid process (7.57% (6.55) and 0.20 mm (0.22)) and at the most distant ramal regions (12.34% (10.19) and 0.14 mm (0.20)) (Table 7 in Supplementary Material 1).

Supplementary Material 1.

## Discussion

The purpose of the present study was to validate and compare the accuracy and reliability of surface- and voxel-based registration on the mandibular rami for long-term 3D evaluation of condylar remodelling. The null hypothesis was: H_0_: the accuracy and reliability of surface- and voxel-based registration on the mandibular rami two years post-surgery are not significantly different in subjects with and without condylar resorption.

Surface-based registration was more accurate and reliable than voxel-based registration on the mandibular ramus two years post-surgery. The measurement error of the repeated condylar volumetric and surface distance measurements using voxel-based registration was 5.44% and 0.52 mm, respectively. This was 3.61% and 0.34 mm larger than for surface-based registration, and is considered a clinical relevant measurement error when assessing the outcome of orthognathic surgery.^45^ In comparison, the measurement error introduced by surface-based registration was below 2% and one voxel (0.3 mm), which is considered clinical irrelevant.

The superior accuracy of surface-based registration was significant for registration of the coronoid process and 9/20 ramal regions in all subjects, including most of the stable reference structures proposed by Verhelst et al.^25^ Surface-based registration was also significantly more accurate for registration of the condyle in subjects without condylar resorption. The superior reliability of surface-based registration was significant for the repeated surface distance measurements of the condyle, the coronoid process and 17/20 ramal regions in all subjects. For the repeated volumetric measurements, the superior reliability was significant at the condyle, at the coronoid process and at 14/20 ramal regions in all subjects. The reliability discrepancy between the two methods was more pronounced in subjects with condylar resorption than in those without.

The ICC range of the repeated measurements of the individual regions using surface-based registration was excellent.^44^ For voxel-based registration, it ranged from poor to excellent. The low ICC and high MAD values of up to 32.19% in volumetric measurement for voxel-based registration were observed at the coronoid process and at the most distant ramal regions with respect to the centre of the applied reference structure. This is in line with the findings of Ruelles et al^36^ and Koerich et al,^41^ who observed that registration errors had a larger effect in areas that are more distant to the reference structure used for the registration. Hence, the repeated registration on the mandibular ramus using the stable reference structures proposed by Verhelst et al^25^ was affected by considerable observer variation. For the most distant ramal regions, the largest accuracy differences between the two methods (MAD value of up to 12.34% in volumetric measurement) were also observed in favour of surface-based registration.

**Figure 6.**
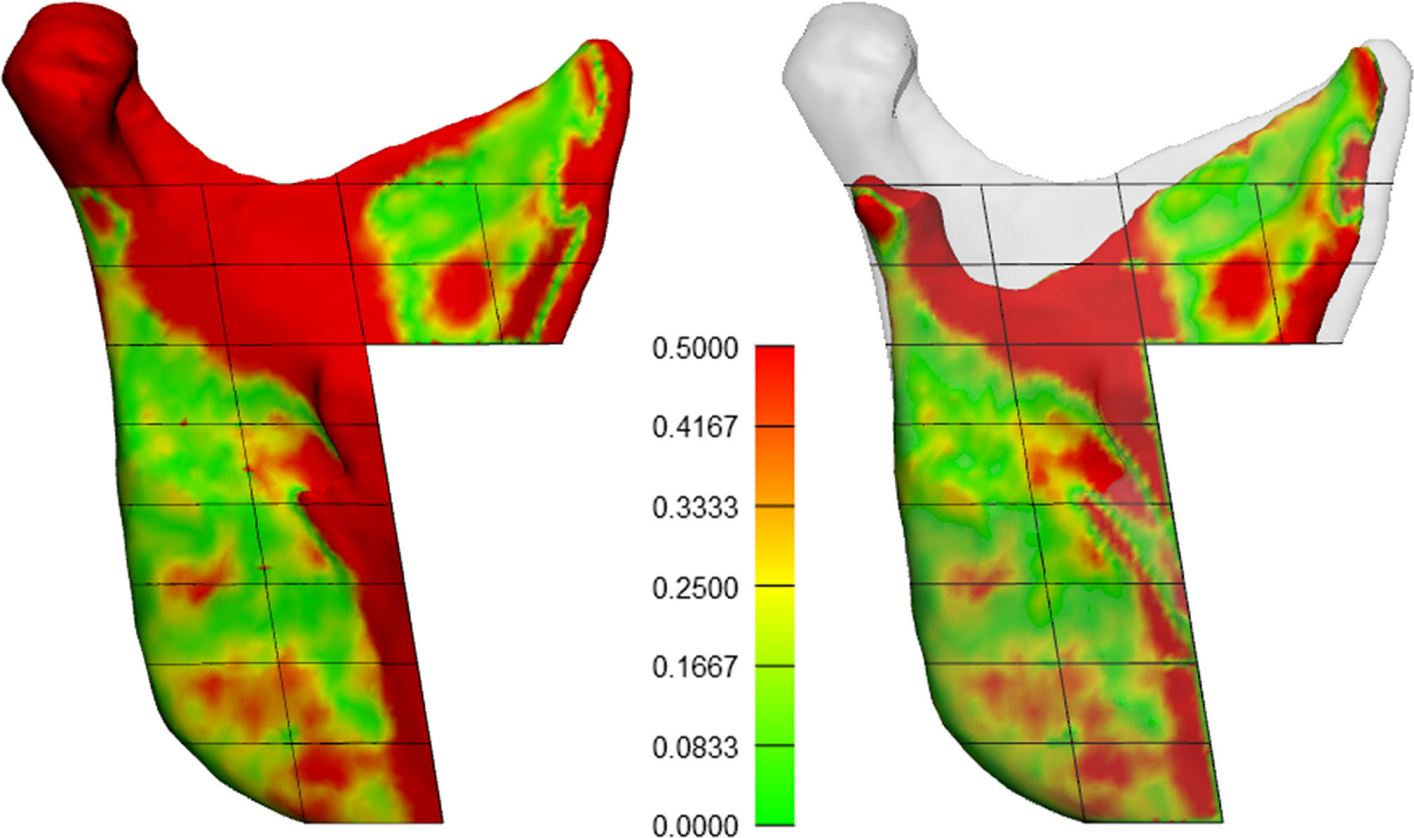
Extreme case of condylar and ramal resorption where voxel-based registration fails using the reference structure proposed by Verhelt et al.^25^ Morphological change shown by color-coded distance map in mm and transparent overlay of the preoperative bone.

Voxel-based registration using the stable reference structures proposed by Verhelst et al^25^ failed in one subject with severe postoperative condylar resorption (Figure 6). Even after several attempts, the registration resulted in a software error or in an obvious misalignment of the postoperative ramal segment. Hence, this subject was removed from the study sample. The reason for this may be explained by the fact that there was not enough mutual image information to match the voxels, and therefore, no adequate control of the 3D transformation.^36,41,46^ However, in the case where voxel-based registration failed, it was possible to perform surface-based registration. Ruellas et al observed similar behaviour of voxel-based registration when evaluating three different reference structures in the distal mandibular segment to register patients’ CBCT scans in growing subjects.^36^ The Slicer software (open-source software by Slicer Community) completed the registration process without errors only in two out of 16 cases using the reference structure suggested by Björk for 2D superimposition.^47^ A mask representing a modified Björk reference structure including more of the surrounding bone, resulted in 14 successful registrations. Finally, the mandibular body was used as reference structure, resulting in all 16 cases being successful, indicating that this reference structure displayed more consistency.^36^ This suggests that when an undersized volume of interest is applied as reference for voxel-based registration, it may fail or produce inconsistent results, especially if that reference structure is not stable.^41,46^

Other reference structures on the mandible have been suggested. Holte et al applied and validated the proximal segment from virtual surgical planning exluding the osteotomy sites as reference structure.^23^ However, as the pupose of this study was 3D assessment of surgical accuracy, the approach was solely validated immediately (two weeks) after surgery.^23^ Schilling et al applied the condyle and condylar neck as reference structure because the mandibular ramus is affected by surgery.^40^ However, when evaluating condylar remodelling, it is very likely that the condyle itself is affected, subsequently unstable and an inappropriate reference structure for registration. Other studies have applied the condylar neck, mandibular notch and the posterior ramal area above the lingula as a reference structure, suggesting that this region is not affected by surgery.^34,37,38^ However, the postoperative stability of these proposed ramal reference structures for image registration using surface-and voxel-based registration has not been validated.

In the present study, the reference structures proposed by Verhelst et al were adopted based on a validation study by the authors.^25^ This study specifically validated a 3D CBCT-based protocol for the follow-up of mandibular condyle remodeling after BSSO using a Obwegeser-Dal Pont split of the ramus with a Hunsuck modification. Hence, this was found to be a suitable method for the present comparative study. However, as the follow-up time was relatively short (six months) and the sample size small (ten subjects), the authors suggested that further studies are needed to confirm that the ramal reference structure does not remodel following surgery.^25^ The findings of the present study indcate that the proposed ramal reference structures, or a part thereof, remodel and affect the performance and applicability of image registration on the ramus for 3D assessment of condylar remodelling, especially in subjects with condylar resorption. The instability of the relatively small reference structure may have compromised the performance of voxel-based registration, as concluded by previous studies.^36,41,46^ In an exhaustive survey on mutual information-based registration of medical images by Plium et al, it was inferred that voxel-based registration may not be a universal cure for all registration problems.^46^ For instance, better results with other measures have been reported for registration of images which show relatively large changes.^46^ In the present comparative study, surface-based registration performed significantly better than voxel-based registration both in terms of accuracy and reliability.

Almukhtar et al concluded that voxel-based registration was associated with less variability, however statistical insignificant, than surface-based registration when using the anterior cranial base as a reference structure.^27^ Han et al concluded that voxel-based registration is more efficient than surface-based registration for CBCT mandibular superimposition.^26^ Surface-based registration requires an extra step compared to voxel-based registration, namely image segmentation and 3D reconstruction of the surface representing the reference structure used for matching, demanding more preprocessing time^26^ and inducing an additional risk of error.^27,41^ On the contrary, voxel-based registration directly matches the CBCT images without the need of image segmentation and surface generation.^20,21^ For 3D assessment of condylar remodelling using volumetric and surface distance measurements, the condyles at a minimum have to be segmented and 3D reconstructed from the pre- and postoperative CBCTs. Hence, the additional segmentation and surface generation of the ramal reference structure can be performed simultaneously, reducing the required time for preprocessing for surface-based registration. Recently, automated image segmentation of the mandible bone using artificial intelligence has been proposed and validated, eliminating the need of manual preprocessing in image segmentation, and thereby eliminating the introduction of additional observer variability.^48–50^

The present comparative study had some limitations. The CBCT images were produced by a single device and was analysed using one, however, certified and widespread, commercial software package - Mimics Innovation Suite (Materialise NV, Leuven, Belgium). Cone-beam computed tomography does not guarantee that a specific threshold in HU in a particular volume corresponds to an identical value in a different volume. However, as the same 3D reconstructed surfaces of the mandibles are applied in the assessment of the compared registration techniques, the potential influence on the outcome is expected to be minor. The characteristics of the 3D surface construction may influence surface-based registration and the 3D objects used in the evaluation of both registration techniques. A low smoothing factor was applied to reduce surface inconsistencies caused by noise with minor influence on the overall object size and shape. Triangle reduction with the specified settings has a minor effect on the object size and shape. However, reduces the number of triangles and memory usage, resulting in a more efficient mesh representation. The BSSO was performed as a Obwegeser-Dal Pont split of the ramus with a Hunsuck modification. No other type of ramus osteotomies were evaluated. No bad splits were present in the data. A bad split may affect the stability of the reference structure, and thereby the registration results. The reference structure used as the region of interest for surface- and voxel-based registration may have remodelled following orthognathic surgery, and thus may have become unstable two years after surgery. This has likely influenced the results of the compared registration techniques, and especially for voxel-based registration. The accuracy of the two registration methods was measured in accordance with previous comparative studies,^26,27^ *i.e*. by the absolute mean surface distance after alignment of the pre- and postoperative rami. As the rami remodel post-surgery, the absolute mean surface distance will most likely never be zero, and thus, this accuracy measure does not represent the ground truth registration result. Remodelling is a confounder that equally affects both registration techniques.

## Conclusion

Surface-based registration was proven more accurate and reliable compared to voxel-based registration on the mandibular ramus two years after Orthognathic Surgery. The measurement error introduced by applying surface-based registration for long-term 3D assessment of condylar remodelling was considered clinical irrelevant, while the one introduced by voxel-based registration was considered clinical relevant. However, importantly, the performance of voxel-based (and surface-based) registration may have been compromised by inappropriate reference structures proposed in the literature.^25^
