# Supplementary material for: Comparison of surface- and voxel-based registration on the mandibular ramus for long-term three-dimensional assessment of condylar remodelling following orthognathic surgery
Source: Dentomaxillofac Radiol. 2022 Feb 25;51(4):20210499. doi: 10.1259/dmfr.20210499 (PMC9499205; doi:10.1259/dmfr.20210499)
Supplement: Supplementary file 1 — Supplementary Material 1. [file dmfr.20210499.supp001.pdf]

Appendix A: Additional results

Table 2. Difference between surface- and voxel-based registration: reliability. Intraclass correlation coefficients (ICC), mean absolute differences (MAD), standard deviations (SD) and paired t-test p-values of the repeated inter-observer condylar and ramal measurements.

Sub-region	Volume difference [%]					Mean surface distance [mm]				
	Surface-based reg.		Voxel-based reg.		P	Surface-based reg.		Voxel-based reg.		P
	ICC	MAD (SD)	ICC	MAD (SD)		ICC	MAD (SD)	ICC	MAD (SD)	
Condyle	1.00	1.37 (1.28)	0.98	3.42 (3.43)	0.009*	0.99	0.10 (0.14)	0.89	0.29 (0.38)	0.013*
Coronoid	0.87	1.96 (2.52)	0.36	10.15 (14.56)	0.010*	0.95	0.04 (0.05)	0.78	0.14 (0.10)	0.001*
Region 1	0.98	1.25 (1.59)	0.90	4.30 (4.41)	0.001*	0.98	0.03 (0.02)	0.88	0.11 (0.11)	0.001*
Region 2	0.97	1.12 (1.51)	0.95	2.06 (2.40)	0.053	0.95	0.02 (0.04)	0.91	0.07 (0.07)	0.008*
Region 3	0.96	0.86 (1.41)	0.73	3.57 (4.23)	0.007*	0.91	0.02 (0.04)	0.83	0.04 (0.07)	0.122
Region 4	0.97	0.80 (0.78)	0.78	2.52 (2.33)	0.004*	0.91	0.01 (0.01)	0.70	0.04 (0.04)	0.004*
Region 5	0.89	1.02 (1.35)	0.74	3.92 (2.91)	0.000*	0.88	0.01 (0.02)	0.50	0.05 (0.06)	0.002*
Region 6	0.85	0.86 (1.16)	0.83	2.72 (2.74)	0.005*	0.93	0.01 (0.01)	0.68	0.04 (0.05)	0.012*
Region 7	0.97	1.19 (0.76)	0.45	5.34 (6.15)	0.005*	0.95	0.02 (0.02)	0.54	0.11 (0.11)	0.002*
Region 8	0.96	4.73 (3.96)	0.35	17.58 (21.76)	0.008*	0.95	0.04 (0.05)	0.41	0.25 (0.33)	0.006*
Region 9	0.92	3.13 (4.42)	0.22	8.44 (11.58)	0.011*	0.89	0.06 (0.08)	0.46	0.10 (0.13)	0.026*
Region 10	0.99	1.11 (1.30)	0.83	3.74 (5.52)	0.013*	0.98	0.03 (0.03)	0.93	0.05 (0.06)	0.014*
Region 11	1.00	0.68 (0.72)	0.98	1.47 (2.10)	0.057	0.98	0.04 (0.06)	0.97	0.06 (0.05)	0.082
Region 12	1.00	0.93 (0.88)	0.97	2.41 (1.91)	0.000*	0.98	0.06 (0.07)	0.96	0.09 (0.08)	0.007*
Region 13	0.82	3.52 (3.62)	0.70	4.44 (4.03)	0.075	0.92	0.07 (0.07)	0.83	0.10 (0.07)	0.012*
Region 14	0.92	2.54 (1.85)	0.84	3.08 (2.33)	0.061	0.94	0.06 (0.06)	0.84	0.09 (0.10)	0.158
Region 15	0.98	1.34 (1.41)	0.93	2.17 (2.87)	0.067	0.93	0.06 (0.08)	0.68	0.13 (0.16)	0.043*
Region 16	0.94	2.67 (1.80)	0.71	5.39 (7.06)	0.034*	0.95	0.06 (0.06)	0.83	0.13 (0.16)	0.046*
Region 17	0.93	1.85 (2.64)	-0.14	8.69 (9.28)	0.004*	0.94	0.02 (0.03)	0.87	0.06 (0.05)	0.007*
Region 18	0.97	1.20 (1.44)	0.62	4.34 (4.98)	0.008*	0.99	0.02 (0.02)	0.91	0.05 (0.06)	0.006*
Region 19	0.99	1.14 (1.45)	0.74	3.21 (5.69)	0.071	0.96	0.03 (0.04)	0.11	0.13 (0.19)	0.013*
Region 20	0.97	2.09 (2.63)	0.58	7.36 (11.27)	0.023*	0.97	0.04 (0.06)	0.51	0.13 (0.21)	0.037*

*Statistically significant.

Table 3. Difference between surface- and voxel-based registration in subjects without condylar resorption: reliability. Intraclass correlation coefficients (ICC), mean absolute differences (MAD), standard deviations (SD) and paired t-test p-values of the repeated inter-observer condylar and ramal measurements.

Sub-region	Volume difference [%]					Mean surface distance [mm]				
	Surface-based reg.		Voxel-based reg.		P	Surface-based reg.		Voxel-based reg.		P
	ICC	MAD (SD)	ICC	MAD (SD)		ICC	MAD (SD)	ICC	MAD (SD)	
Condyle	0.97	0.92 (0.50)	0.95	1.40 (1.06)	0.061	0.95	0.02 (0.03)	0.89	0.06 (0.04)	0.025*
Coronoid	0.89	1.17 (1.50)	0.58	4.61 (1.73)	0.001*	0.64	0.03 (0.04)	0.26	0.15 (0.07)	0.000*
Region 1	0.98	0.97 (0.66)	0.91	1.68 (1.21)	0.094	0.97	0.02 (0.01)	0.75	0.05 (0.04)	0.013*
Region 2	0.99	0.48 (0.37)	0.98	0.96 (0.80)	0.031*	0.99	0.01 (0.01)	0.95	0.02 (0.02)	0.016*
Region 3	0.99	0.26 (0.31)	0.96	1.33 (0.96)	0.001*	0.99	0.01 (0.00)	0.91	0.02 (0.03)	0.049*
Region 4	0.99	0.52 (0.36)	0.93	1.86 (1.21)	0.001*	0.98	0.01 (0.01)	0.90	0.02 (0.02)	0.020*
Region 5	0.91	1.11 (1.62)	0.79	2.55 (1.76)	0.002*	0.90	0.01 (0.03)	0.79	0.04 (0.04)	0.043*
Region 6	0.93	0.74 (0.56)	0.64	1.59 (1.28)	0.071	0.97	0.01 (0.01)	0.64	0.04 (0.05)	0.037*
Region 7	0.61	1.07 (0.78)	0.64	2.18 (2.14)	0.101	0.95	0.02 (0.02)	0.59	0.05 (0.07)	0.066
Region 8	0.90	3.22 (3.37)	0.96	2.96 (1.62)	0.580	0.97	0.02 (0.04)	0.88	0.06 (0.07)	0.096
Region 9	0.99	0.88 (0.77)	0.97	1.62 (1.36)	0.045*	0.98	0.02 (0.03)	0.96	0.03 (0.02)	0.176
Region 10	0.99	0.77 (1.00)	0.99	0.84 (0.81)	0.413	0.99	0.02 (0.01)	0.99	0.02 (0.01)	0.347
Region 11	0.99	0.90 (0.92)	0.99	0.77 (0.90)	0.800	0.93	0.06 (0.07)	0.94	0.06 (0.06)	0.660
Region 12	0.99	0.82 (0.81)	0.99	1.63 (0.98)	0.018*	0.84	0.07 (0.08)	0.81	0.09 (0.08)	0.040*
Region 13	0.52	3.73 (3.66)	0.63	3.66 (3.57)	0.632	0.80	0.07 (0.09)	0.71	0.08 (0.09)	0.132
Region 14	0.78	2.05 (2.09)	0.83	1.87 (1.89)	0.879	0.98	0.03 (0.04)	0. 81	0.07 (0.09)	0.098
Region 15	0.50	1.29 (1.74)	0.43	1.42 (2.18)	0.337	0.99	0.03 (0.03)	0.61	0.09 (0.11)	0.066
Region 16	0.63	2.51 (2.00)	0.76	2.09 (1.90)	0.810	0.98	0.04 (0.03)	0.71	0.09 (0.10)	0.072
Region 17	0.93	1.24 (1.29)	0.81	2.47 (0.99)	0.053	0.90	0.01 (0.01)	0.55	0.06 (0.04)	0.004*
Region 18	0.96	1.21 (1.44)	0.97	1.46 (0.84)	0.250	1.00	0.01 (0.01)	0.95	0.04 (0.05)	0.066
Region 19	0.97	0.97 (1.26)	0.94	1.75 (1.04)	0.059	0.97	0.01 (0.01)	0.00	0.07 (0.11)	0.049*
Region 20	0.99	0.81 (0.94)	0.92	2.20 (1.94)	0.009*	0.98	0.03 (0.05)	0.91	0.06 (0.08)	0.161

*Statistically significant.

Table 4. Difference between surface- and voxel-based registration in subjects with condylar resorption: reliability. Intraclass correlation coefficients (ICC), mean absolute differences (MAD), standard deviations (SD) and paired t-test p-values of the repeated inter-observer condylar and ramal measurements.

Sub-region	Volume difference [%]					Mean surface distance [mm]				
	Surface-based reg.		Voxel-based reg.		P	Surface-based reg.		Voxel-based reg.		P
	ICC	MAD (SD)	ICC	MAD (SD)		ICC	MAD (SD)	ICC	MAD (SD)	
Condyle	0.99	1.83 (1.66)	0.95	5.44 (3.82)	0.015*	0.98	0.18 (0.16)	0.79	0.52 (0.43)	0.020*
Coronoid	0.81	2.76 (3.12)	0.23	15.69 (19.40)	0.034*	0.95	0.05 (0.06)	0.87	0.13 (0.13)	0.091
Region 1	0.96	1.52 (2.17)	0.86	6.93 (4.93)	0.002*	0.97	0.03 (0.03)	0.84	0.17 (0.13)	0.002*
Region 2	0.95	1.77 (1.93)	0.93	3.17 (2.97)	0.116	0.92	0.04 (0.05)	0.87	0.11 (0.08)	0.024*
Region 3	0.93	1.45 (1.82)	0.58	5.82 (5.06)	0.024*	0.86	0.04 (0.05)	0.77	0.07 (0.10)	0.244
Region 4	0.91	1.07 (1.00)	0.55	3.19 (3.00)	0.047*	0.85	0.02 (0.02)	0.51	0.05 (0.05)	0.029*
Region 5	0.81	0.92 (1.09)	0.73	5.28 (3.26)	0.003*	0.69	0.01 (0.01)	0.10	0.07 (0.07)	0.014*
Region 6	0.80	0.98 (1.58)	0.85	3.85 (3.37)	0.015*	0.79	0.02 (0.02)	0.62	0.04 (0.05)	0.108
Region 7	0.97	1.31 (0.76)	0.22	8.50 (7.29)	0.009*	0.92	0.03 (0.03)	0.23	0.16 (0.12)	0.005*
Region 8	0.96	6.25 (4.08)	0.01	32.19 (22.85)	0.004*	0.91	0.06 (0.06)	0.10	0.45 (0.38)	0.007*
Region 9	0.83	5.38 (5.42)	-0.14	15.26 (13.34)	0.015*	0.76	0.09 (0.10)	0.17	0.18 (0.15)	0.036*
Region 10	0.98	1.45 (1.52)	0.73	6.65 (6.70)	0.011*	0.96	0.04 (0.04)	0.87	0.09 (0.07)	0.013*
Region 11	1.00	0.46 (0.38)	0.97	2.16 (2.72)	0.040*	1.00	0.02 (0.03)	0.98	0.06 (0.04)	0.045*
Region 12	0.99	1.04 (0.97)	0.95	3.18 (2.33)	0.002*	0.99	0.05 (0.06)	0.97	0.08 (0.07)	0.035*
Region 13	0.87	3.31 (3.76)	0.71	5.23 (4.49)	0.066	0.93	0.07 (0.04)	0.78	0.12 (0.05)	0.025*
Region 14	0.93	3.02 (1.52)	0.84	4.30 (2.15)	0.029*	0.87	0.09 (0.07)	0.78	0.11 (0.10)	0.342
Region 15	0.99	1.38 (1.08)	0.94	2.93 (3.38)	0.079	0.87	0.10 (0.10)	0.51	0.17 (0.19)	0.152
Region 16	0.96	2.83 (1.68)	0.62	8.68 (8.80)	0.019*	0.93	0.09 (0.08)	0.78	0.18 (0.20)	0.133
Region 17	0.93	2.46 (3.50)	-0.52	14.91 (9.73)	0.005*	0.94	0.04 (0.05)	0.92	0.07 (0.05)	0.133
Region 18	0.97	1.19 (1.51)	0.41	7.22 (5.76)	0.008*	0.98	0.02 (0.02)	0.85	0.06 (0.06)	0.030*
Region 19	0.99	1.30 (1.67)	0.65	4.68 (7.91)	0.119	0.94	0.05 (0.05)	-0.19	0.19 (0.24)	0.053
Region 20	0.96	3.38 (0.00)	0.44	12.51 (14.33)	0.042*	0.96	0.05 (0.07)	0.26	0.21 (0.28)	0.063

*Statistically significant.

Table 5. Difference between surface- and voxel-based registration: accuracy. Intraclass correlation coefficients (ICC), mean absolute differences (MAD), standard deviations (SD), paired t-test p-values and 95% confidence intervals of the condylar and ramal measurements.

Sub-region	Volume difference [%]				Mean surface distance [mm]			
	ICC	MAD (SD)	P	95% conf. interval	ICC	MAD (SD)	P	95% conf. interval
Condyle	0.99	1.93 (1.73)	0.347	[-1.25 – 0.45]	0.93	0.21 (0.28)	0.973	[-0.11 – 0.12]
Coronoid	0.66	6.99 (5.36)	0.006*	[-6.43 – -1.16]	0.52	0.21 (0.23)	0.000*	[-0.27 – -0.10]
Region 1	0.83	3.24 (4.57)	0.034*	[-3.63 – -0.15]	0.78	0.07 (0.11)	0.003*	[-0.10 – -0.02]
Region 2	0.84	2.87 (3.05)	0.000*	[-3.61 – -1.39]	0.80	0.06 (0.08)	0.000*	[-0.08 – -0.03]
Region 3	0.78	2.84 (2.23)	0.000*	[-3.21 – -1.37]	0.78	0.05 (0.05)	0.000*	[-0.06 – -0.03]
Region 4	0.58	2.57 (2.79)	0.003*	[-2.86 – -0.64]	0.32	0.05 (0.05)	0.000*	[-0.06 – -0.03]
Region 5	0.74	3.05 (3.57)	0.188	[-2.52 – 0.51]	0.65	0.05 (0.05)	0.000*	[-0.07 – -0.03]
Region 6	0.62	3.04 (3.77)	0.008*	[-3.46 – -0.54]	0.66	0.07 (0.06)	0.000*	[-0.08 – -0.04]
Region 7	0.81	3.21 (2.97)	0.006*	[-3.19 – -0.58]	0.79	0.07 (0.07)	0.000*	[-0.09 – -0.04]
Region 8	0.75	10.05 (8.61)	0.132	[-7.48 – 1.02]	0.80	0.10 (0.11)	0.151	[-0.08 – 0.01]
Region 9	0.74	5.09 (6.91)	0.491	[-1.85 – 3.78]	0.67	0.08 (0.11)	0.932	[-0.05 – 0.04]
Region 10	0.93	1.85 (3.41)	0.768	[-1.47 – 1.09]	0.94	0.04 (0.05)	0.229	[-0.04 – 0.01]
Region 11	0.99	1.10 (1.17)	0.023*	[-1.07 – -0.08]	0.98	0.04 (0.04)	0.024*	[-0.04 – 0.00]
Region 12	0.99	1.31 (1.06)	0.546	[-0.73 – 0.39]	0.98	0.05 (0.05)	0.519	[-0.03 – 0.01]
Region 13	0.98	1.04 (1.15)	0.769	[-0.44 – 0.59]	0.97	0.04 (0.04)	0.327	[-0.03 – 0.01]
Region 14	0.97	1.11 (1.72)	0.871	[-0.73 – 0.62]	0.85	0.07 (0.09)	0.385	[-0.05 – 0.02]
Region 15	0.97	1.37 (1.64)	0.890	[-0.66 – 0.75]	0.68	0.10 (0.15)	0.234	[-0.09 – 0.02]
Region 16	0.91	2.59 (2.67)	0.976	[-1.25 – 1.21]	0.72	0.12 (0.16)	0.551	[-0.08 – 0.05]
Region 17	0.41	5.72 (5.03)	0.040*	[-4.88 – -0.12]	0.54	0.09 (0.09)	0.001*	[-0.10 – -0.03]
Region 18	0.91	2.21 (2.08)	0.061	[-1.87 – 0.05]	0.77	0.06 (0.09)	0.262	[-0.05 – 0.02]
Region 19	0.91	3.18 (3.66)	0.041*	[0.07 – 3.10]	0.58	0.12 (0.15)	0.999	[-0.06 – 0.06]
Region 20	0.89	6.08 (5.09)	0.160	[-0.75 – 4.37]	0.68	0.12 (0.14)	0.163	[-0.02 – 0.10]

*Statistically significant.

Table 6. Difference between surface- and voxel-based registration in subjects without condylar resorption: accuracy. Intraclass correlation coefficients (ICC), mean absolute differences (MAD), standard deviations (SD), paired t-test p-values and 95% confidence intervals of the condylar and ramal measurements.

Sub-region	Volume difference [%]				Mean surface distance [mm]			
	ICC	MAD (SD)	P	95% conf. interval	ICC	MAD (SD)	P	95% conf. interval
Condyle	0.94	1.41 (1.11)	0.002*	[-1.79 – -0.47]	0.60	0.11 (0.08)	0.000*	[-0.14 – -0.05]
Coronoid	0.67	6.47 (4.11)	0.055	[-6.54 – 0.07]	0.27	0.22 (0.25)	0.002*	[-0.33 – -0.09]
Region 1	0.88	1.95 (1.34)	0.563	[-1.43 – 0.80]	0.87	0.03 (0.03)	0.001*	[-0.05 – -0.01]
Region 2	0.90	1.79 (1.21)	0.004*	[-2.10 – -0.45]	0.87	0.03 (0.03)	0.000*	[-0.05 – -0.02]
Region 3	0.70	2.67 (2.10)	0.001*	[-3.43 – -0.99]	0.57	0.05 (0.05)	0.001*	[-0.07 – -0.02]
Region 4	0.65	2.62 (3.05)	0.156	[-3.07 – 0.53]	0.36	0.04 (0.05)	0.001*	[-0.07 – -0.02]
Region 5	0.91	2.37 (2.30)	0.487	[-2.07 – 1.02]	0.76	0.05 (0.04)	0.000*	[-0.07 – -0.03]
Region 6	0.92	1.78 (1.20)	0.113	[-1.72 – 0.20]	0.53	0.06 (0.05)	0.000*	[-0.08 – -0.03]
Region 7	0.67	2.98 (2.54)	0.009*	[-3.69 – -0.59]	0.66	0.06 (0.04)	0.000*	[-0.08 – -0.03]
Region 8	0.53	7.98 (6.46)	0.046*	[-8.86 – -0.09]	0.78	0.08 (0.07)	0.243	[-0.08 – 0.02]
Region 9	0.94	2.58 (1.99)	0.080	[-2.69 – 0.16]	0.88	0.05 (0.04)	0.019*	[-0.05 – -0.01]
Region 10	0.99	1.14 (1.12)	0.061	[-1.34 – 0.03]	0.98	0.02 (0.02)	0.004*	[-0.03 – -0.01]
Region 11	0.99	0.89 (0.97)	0.112	[-1.04 – 0.12]	0.98	0.03 (0.03)	0.004*	[-0.04 – -0.01]
Region 12	0.99	1.26 (1.11)	0.329	[-1.15 – 0.41]	0.97	0.04 (0.03)	0.146	[-0.04 – 0.01]
Region 13	1.00	0.64 (0.61)	0.228	[-0.64 – 0.16]	0.98	0.04 (0.03)	0.426	[-0.03 – 0.01]
Region 14	0.99	0.79 (0.61)	0.402	[-0.66 – 0.27]	0.91	0.06 (0.08)	0.910	[-0.04 – 0.05]
Region 15	0.96	1.26 (1.09)	0.663	[-0.62 – 0.95]	0.49	0.08 (0.13)	0.976	[-0.07 – 0.08]
Region 16	0.74	3.02 (3.20)	0.598	[-1.54 – 2.60]	0.74	0.10 (0.10)	0.405	[-0.04 – 0.09]
Region 17	0.28	4.66 (3.76)	0.016*	[-5.51 – -0.64]	0.02	0.10 (0.11)	0.002*	[-0.14 – -0.04]
Region 18	0.92	2.08 (1.43)	0.351	[-1.71 – 0.64]	0.77	0.06 (0.10)	0.117	[-0.09 – 0.01]
Region 19	0.94	2.40 (1.68)	0.489	[-0.91 – 1.84]	0.16	0.10 (0.17)	0.257	[-0.14 – 0.04]
Region 20	0.58	5.19 (3.73)	0.169	[-0.92 – 4.87]	0.71	0.11 (0.14)	0.967	[-0.08 – 0.08]

*Statistically significant.

Table 7. Difference between surface- and voxel-based registration in subjects with condylar resorption: accuracy. Intraclass correlation coefficients (ICC), mean absolute differences (MAD), standard deviations (SD), paired t-test p-values and 95% confidence intervals of the condylar and ramal measurements.

Sub-region	Volume difference [%]				Mean surface distance [mm]			
	ICC	MAD (SD)	P	95% conf. interval	ICC	MAD (SD)	P	95% conf. interval
Condyle	0.99	2.52 (2.11)	0.602	[-1.23 – 2.06]	0.89	0.34 (0.37)	0.353	[-0.13 – 0.35]
Coronoid	0.61	7.57 (6.55)	0.055	[-8.94 – 0.11]	0.64	0.20 (0.22)	0.027*	[-0.28 – -0.02]
Region 1	0.76	4.67 (6.27)	0.041*	[-7.10 – -0.17]	0.72	0.11 (0.14)	0.027*	[-0.17 – -0.01]
Region 2	0.81	4.08 (3.97)	0.001*	[-5.94 – -1.77]	0.75	0.10 (0.10)	0.003*	[-0.14 – -0.03]
Region 3	0.80	3.02 (2.42)	0.004*	[-3.92 – -0.85]	0.82	0.06 (0.04)	0.000*	[-0.08 – -0.03]
Region 4	0.45	2.52 (2.56)	0.003*	[-3.67 – -0.90]	0.28	0.05 (0.05)	0.002*	[-0.07 – -0.02]
Region 5	0.21	3.80 (4.54)	0.275	[-4.41 – 1.34]	0.52	0.06 (0.06)	0.002*	[-0.08 – -0.02]
Region 6	0.38	4.44 (5.04)	0.026*	[-6.29 – -0.47]	0.71	0.08 (0.07)	0.003*	[-0.11 – -0.03]
Region 7	0.81	3.47 (3.44)	0.164	[-3.93 – 0.72]	0.78	0.09 (0.09)	0.015*	[-0.12 – -0.02]
Region 8	0.74	12.34 (10.19)	0.633	[-9.90 – 6.19]	0.76	0.13 (0.14)	0.347	[-0.13 – 0.05]
Region 9	0.58	7.87 (9.16)	0.228	[-2.37 – 9.27]	0.46	0.12 (0.15)	0.523	[-0.06 – 0.12]
Region 10	0.84	2.65 (4.76)	0.801	[-2.39 – 3.05]	0.85	0.07 (0.07)	0.749	[-0.06 – 0.04]
Region 11	0.99	1.33 (1.35)	0.110	[-1.59 – 0.18]	0.97	0.05 (0.06)	0.340	[-0.06 – 0.02]
Region 12	0.99	1.37 (1.04)	0.886	[-0.81 – 0.93]	0.98	0.05 (0.05)	0.836	[-0.03 – 0.04]
Region 13	0.96	1.49 (1.43)	0.392	[-0.59 – 1.44]	0.92	0.05 (0.05)	0.539	[-0.05 – 0.03]
Region 14	0.94	1.46 (2.40)	0.887	[-1.31 – 1.51]	0.76	0.08 (0.11)	0.246	[-0.10 – 0.03]
Region 15	0.97	1.50 (2.12)	0.896	[-1.39 – 1.22]	0.71	0.12 (0.17)	0.124	[-0.17 – 0.02]
Region 16	0.97	2.11 (1.89)	0.355	[-2.02 – 0.77]	0.71	0.14 (0.20)	0.229	[-0.19 – 0.05]
Region 17	0.42	6.90 (6.03)	0.397	[-6.40 – 2.67]	0.75	0.09 (0.08)	0.205	[-0.09 – 0.02]
Region 18	0.91	2.35 (2.67)	0.108	[-2.99 – 0.33]	0.77	0.06 (0.08)	0.941	[-0.05 – 0.05]
Region 19	0.90	4.06 (4.94)	0.053	[-0.05 – 5.70]	0.70	0.14 (0.12)	0.197	[-0.03 – 0.14]
Region 20	0.90	7.06 (6.25)	0.475	[-3.07 – 6.32]	0.61	0.14 (0.14)	0.046*	[0.00 – 0.18]

*Statistically significant.
